# 3/2 fractional quantum Hall plateau in confined two-dimensional electron gas

**DOI:** 10.1038/s41467-019-12245-y

**Published:** 2019-09-25

**Authors:** Hailong Fu, Yijia Wu, Ruoxi Zhang, Jian Sun, Pujia Shan, Pengjie Wang, Zheyi Zhu, L. N. Pfeiffer, K. W. West, Haiwen Liu, X. C. Xie, Xi Lin

**Affiliations:** 1 International Center for Quantum Materials, Peking University, 100871 Beijing, China; 2 Department of Physics, The Pennsylvania State University, University Park, PA 16802 USA; 3 Department of Electrical Engineering, Princeton University, Princeton, NJ 08544 USA; 4 Center for Advanced Quantum Studies, Department of Physics, Beijing Normal University, 100875 Beijing, China; 5 Beijing Academy of Quantum Information Sciences, 100193 Beijing, China; 6 CAS Center for Excellence in Topological Quantum Computation, University of Chinese Academy of Sciences, 100190 Beijing, China

**Keywords:** Phase transitions and critical phenomena, Quantum Hall

## Abstract

Even-denominator fractional quantum Hall (FQH) states, such as 5/2 and 7/2, have been well known in a two-dimensional electron gas (2DEG) for decades and are still investigated as candidates of non-Abelian statistics. In this paper, we present the observation of a 3/2 FQH plateau in a single-layer 2DEG with lateral confinement at a bulk filling factor of 5/3. The 3/2 FQH plateau is quantized at $\left( {\frac{h}{{e^2}}} \right)/\left( {\frac{3}{2}} \right)$ within 0.02%, and can survive up to 300 mK. This even-denominator FQH plateau may imply intriguing edge structure and excitation in FQH system with lateral confinement. The observations in this work demonstrate that understanding the effect of the lateral confinement on the many-body system is critical in the pursuit of important theoretical proposals involving edge physics, such as the demonstration of non-Abelian statistics and the realization of braiding for fault-tolerant quantum computation.

## Abstract (web summary)

Fractional quantum Hall states in 2D electron gases arise due to strong electron-electron interactions, which makes a general theoretical understanding difficult. Fu et al. present data showing the *ν* = 5/3 quantum Hall state has a 3/2 plateau in the diagonal resistance that has not been captured by existing models.

## Introduction

The fractional quantum Hall (FQH) effect has been investigated to extend our understanding of strongly interacting particles in two dimensions since its discovery^1,2^. Most of the observed fractional states are odd-denominator states^3^ and can be explained under the framework of the composite fermion theory^4^. For example, the 1/3 FQH state can be mapped into the filling factor *ν* = 1 integer quantum Hall (IQH) state of composite fermions carrying fractional charge of e/3, which leads to a fractional $\frac{1}{3}\frac{{e^2}}{h}$ Hall conductance. At half-filled *N* = 0 Landau level (*ν* = 1/2, 3/2), the absence of FQH states is attributed to the formation of Fermi sea of composite fermions^4,5^. The discovery of the first even-denominator state, the 5/2 FQH state^6,7^, viewed as a paired state of composite fermions with e/4 fractional charge^8^, further enriched the knowledge of interacting electrons. Recent studies such as phase transition to the stripe phase^9^, and even-denominator states in other two-dimensional systems^10,11^, keep providing interesting physics at even-denominator filling factors.

The excitations in the FQH system carry fractional charge owing to the strong correlation between electrons. Besides, there is another equally famous way of charge fractionalization through topological soliton and topological phase transition^12–14^, which is related to the boundary of a realistic experimental system. Theoretical treatments for the FQH effect usually consider an infinite 2DEG, but experiments can only measure finite samples with boundaries. The quantum Hall edge originating from the boundary was discussed^15^ and later described by chiral Luttinger liquids^16^. Experiments based on the FQH edge physics have provided many exciting results and led to more efforts, such as the measurement of fractional charge^17^, weak quasi-particle tunneling between edge currents^18,19^ and the search of the neutral modes^20^. Theoretically, a 2DEG confined in an interferometer is proposed to test the non-Abelian statistics directly^21^, and the interference of the edge current has been measured^22^. Although the realistic boundary of a many-body system may complicate the theoretical treatment^23^, the confinement can be a powerful experimental approach to manipulate the system. Even though the FQH effect has been studied for decades, systems of interacting electrons with confinement are still an open area for exploration.

Here we show an even-denominator FQH plateau quantized at $\left( {\frac{h}{{e^2}}} \right)/\left( {\frac{3}{2}} \right)$under lateral confinement in a single-layer 2DEG. This plateau develops below 300 mK with a quantization of 0.02%. The current transmitting through the confined region also causes a quantized two-terminal conductance at $\frac{3}{2}\frac{{e^2}}{h}$, and a backscattered current induces a quantized tunneling conductance at $\frac{1}{6}\frac{{e^2}}{h}$.

## Results

### Gate-defined confinement

**Fig. 1:**
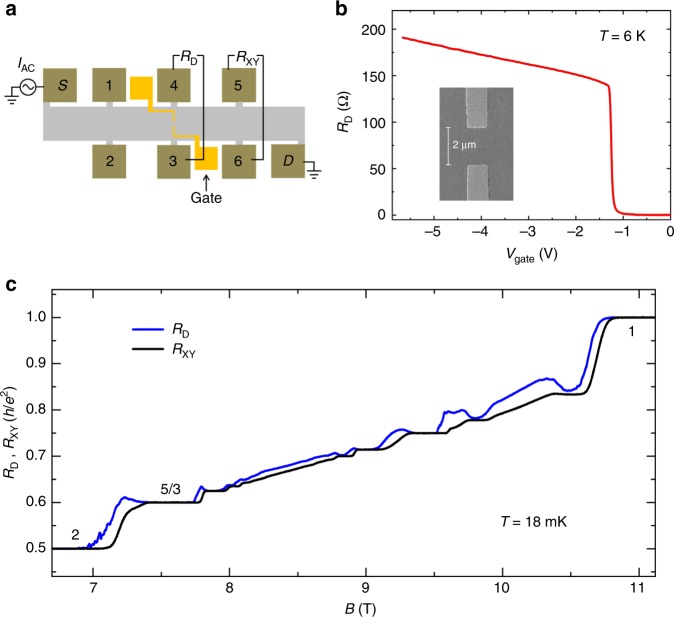
Sample information and the diagonal resistance and the Hall resistance traces. **a** A sketch of the Hall bar and the measurement setup. **b** Gate voltage dependence of the diagonal resistance *R*_D_ at B = 0 T and *T* = 6 K. The inset is an SEM picture of a device with the same gate geometry as that used in this experiment. The lithography dimension is 1 × 2 μm^2^. **c** The diagonal resistance and the Hall resistance traces with *V*_gate_ = −4.5 V at 18 mK. The black line is the Hall resistance *R*_XY_ and the blue line is the diagonal resistance *R*_D_. Both of them develop a series of IQH and FQH plateaus. *R*_D_ was measured from contact 3 to contact 4, and *R*_XY_ was from contact 6 to contact 5. Source data are provided as a Source Data file

The Hall bar samples were made from a wafer of GaAs/AlGaAs heterostructures. The 2DEG is about 200 nm below the sample surface. Two separate gates were deposited on the surface for the confined region. A sketch of the Hall bar and measurement setup are shown in Fig. 1a. Fig. 1b shows the gate voltage dependence of the diagonal resistance *R*_D_ across the confined region at zero magnetic field and *T* = 6 K. *R*_D_ increases quickly from near zero to about 140 Ω when the gate voltage is swept from −1.0 V to −1.3 V, and below −1.3 V *R*_D_ increases slowly, indicating the electrons underneath the gates are depleted when the gate voltage is not positive than −1.3 V. By estimating the transverse modes through the confined region, 140 Ω corresponds to a lateral confinement of ~2.1 μm, close to the lithography length. In order to achieve the confined region, *V*_gate_ = −4.5 V was applied to the gates to deplete the electrons underneath and around the gates. This gate voltage was kept for more than 6 h above 4 K and during the cooling to 18 mK. This procedure named gate annealing is similar to that used in quasi-particle tunneling experiments for uniform density^18,19,24,25^. At 18 mK, we kept gate voltage at −4.5 V, and measured the Hall traces between filling factor 1 and 2. Both the Hall resistance *R*_XY_ in the bulk of the Hall bar and the diagonal resistance *R*_D_ develop two IQH plateaus and several FQH plateaus, which indicates that well-defined compressible edge states and incompressible bulk states appear in the confined region, and the density inside the confined region and outside the confined region is nearly uniform (Fig. 1c).

### Four-terminal resistance

**Fig. 2:**
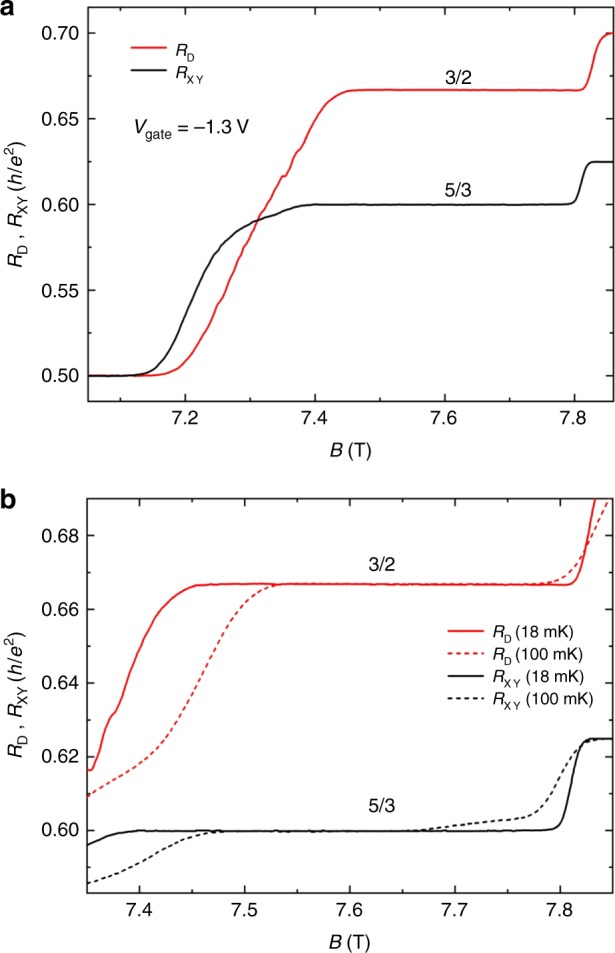
The diagonal resistance and the Hall resistance traces around the *ν* = 5/3 FQH state with *V*_gate_ = −1.3 V. **a** At 18 mK, the Hall resistance (the black line) is quantized at $\left( {\frac{h}{{e^2}}} \right)/\left( {\frac{5}{3}} \right)$, and the diagonal resistance (the red line) is quantized at $\left( {\frac{h}{{e^2}}} \right)/\left( {\frac{3}{2}} \right)$. **b** Temperature dependence of the 3/2 FQH plateau of the diagonal resistance and the 5/3 FQH plateau of the Hall resistance in the bulk of the Hall bar. Source data are provided as a Source Data file

In our samples, the confined region is formed and controlled by the gate voltages. Changing the gate voltage will tune the lateral confinement but not influence the bulk properties. As shown in Fig. 2a, when the gate voltage is changed to −1.3 V, the Hall resistance *R*_XY_ does not change. Unexpectedly, the diagonal resistance *R*_D_ develops an even-denominator FQH plateau quantized at $\left( {\frac{h}{{e^2}}} \right)/\left( {\frac{3}{2}} \right)$, with a quantization of 0.02%, the resolution of this experiment. The 3/2 FQH plateau of *R*_D_ and the 5/3 FQH plateau of *R*_XY_ center at almost the same magnetic field, and the width of these two plateaus are similar at 18 mK. Analogous to conventional FQH states, the width of the 3/2 plateau decreases with increasing temperature. Interestingly, the 3/2 FQH plateau is even wider than that of the 5/3 FQH plateau at 100 mK (Fig. 2b), and the 3/2 FQH plateau can survive up to 300 mK (Supplementary Fig. 1), which indicates that the 3/2 plateau is more stable than all the previously observed even-denominator FQH plateaus in a single-layer 2DEG. The observation of such a 3/2 FQH plateau has been reproduced in different samples with different device sizes (see Supplementary Note 1).

**Fig. 3:**
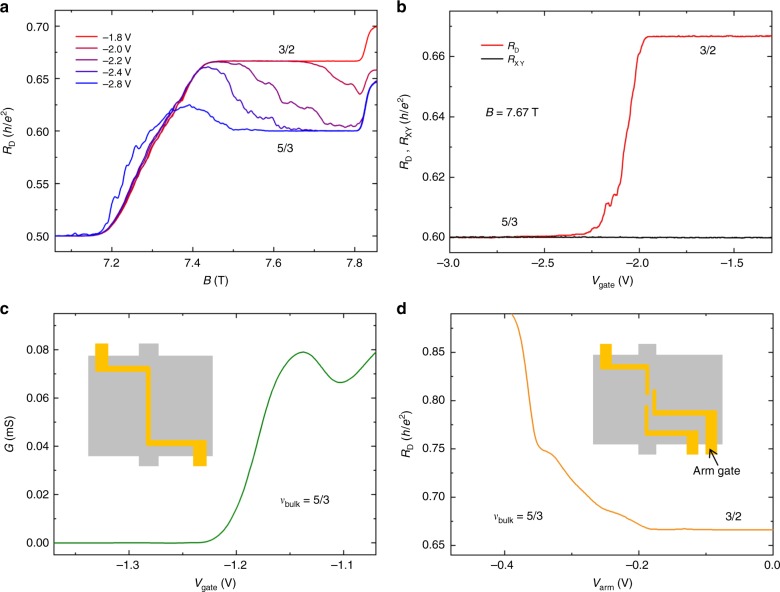
Gate voltage dependence of the diagonal resistance, the Hall resistance and the two-terminal conductance. **a** Magnetic field dependence of the diagonal resistance with different gate voltages. **b** Gate voltage dependence of the diagonal resistance and the Hall resistance at 7.67 T. The gate voltage was changed from −1.3 V to −3.0 V. **c** Gate voltage dependence of the two-terminal conductance across the single top gate. The gate was annealed at −4.5 V. The conductance was measured at bulk filling factor 5/3. The measurement was carried out by applying a voltage excitation to the source contact and measuring the current from drain contact at the other side of the mesa. The inset is the sketch of the device and the device was made from the same wafer as that used in **a**. The minimum width of the top gate is 1.5 μm, the same width as that of the gates used in one sample, with which the 3/2 plateau is observed (Supplementary Fig. 2). **d** Arm gate voltage (*V*_arm_) dependence of the diagonal resistance at bulk filling factor 5/3. The confinement gates were annealed at −4.5 V. The arm gate was not annealed. During the measurement the confinement gates were kept at −1.3 V. The inset is the sketch of the device and the device was made from the same wafer as that used in **a**. The dimension of the confinement is 1 × 2 μm^2^. All these measurements were performed at 18 mK. Source data are provided as a Source Data file

The critical parameter to trigger the appearance of the 3/2 FQH plateau is the gate voltage. The diagonal resistance traces with different gate voltages at 18 mK are shown in Fig. 3a. The diagonal resistance develops the 5/3 FQH plateau with *V*_gate_ = −2.8 V, and becomes larger with less negative gate voltage. When the gate voltage is less negative than or equal to −2.0 V, the 3/2 FQH plateau appears. The gate voltage dependence of the 3/2 FQH plateau can also be demonstrated by the relationship between the gate voltage and the diagonal resistance. As shown in Fig. 3b, the diagonal resistance changes from $\left( {\frac{h}{{e^2}}} \right)/\left( {\frac{3}{2}} \right)$ to $\left( {\frac{h}{{e^2}}} \right)/\left( {\frac{5}{3}} \right)$ when the gate voltage is swept from −1.3 V to −3.0 V at the magnetic field of 7.67T, near the center of the 5/3 FQH plateau in the bulk. At the same time, the Hall resistance is consistently quantized at $\left( {\frac{h}{{e^2}}} \right)/\left( {\frac{5}{3}} \right)$, indicating the 5/3 FQH state in the bulk of the Hall bar is not influenced by the gate voltage.

Another essential condition for the observation of the 3/2 FQH plateau is the gate annealing procedure with appropriate negative gate voltage. In our samples, *V*_gate_ = −1.3 V is sufficient for the depletion of the electrons underneath the gates, as shown in Fig. 1b and Fig. 3c. During the measurements for the 3/2 FQH plateaus in Fig. 2, Fig. 3a, b, we did the annealing procedure with *V*_gate_ = −4.5 V. If the gates are annealed at -1.5 V, even though enough to achieve the confined region, the 3/2 FQH plateau will not appear (Supplementary Fig. 2).

In order to demonstrate the full depletion underneath the confinement gates, we fabricated a sample with a single top gate crossing the entire Hall bar. This gate was also annealed at *V*_gate_ = −4.5 V, and the measurement was carried out at bulk filling factor 5/3 and *T* = 18 mK. As shown in Fig. 3c, the conductance across the top gate is always zero when the gate voltage is more negative than −1.25 V. In another sample, we fabricated an individual gate (defined as arm gate in Fig. 3d) next to the confined region. As shown in Fig. 3d, a small negative gate voltage on this arm gate, far away from depleting values, is enough to break the 3/2 FQH plateau. These two control experiments demonstrated that the 3/2 FQH plateau is induced by the confined region.

In the confined region, a possible result caused by the annealing procedure and gate voltage change is quasi-particle tunneling. When *V*_gate_ = −4.5 V is applied to the gates, the electrons both underneath and around the gates will be depleted. The annealing procedure may adjust the charge distribution of the donor layer at *T* > 4 K, which causes a very sharp potential in the confined region at the measurement temperature range, and supports the 5/3 FQH state in the confined region (Fig. 1c). When the gate voltage is changed to less negative values, such as −2.0 V in Fig. 3a, at the lower temperature, the effective residual potential from both the gates and the donor layer may generate some disorders to the 2DEG, which facilitate the quasi-particle tunneling between the counter-propagating edge states in the confined region. More negative annealing gate voltage and less negative measurement gate voltage will generate more disorders, resulting in stronger quasi-particle tunneling. Strictly, the diagonal resistance is the sum of the Hall resistance in the bulk of the Hall bar and the tunneling resistance in the confined region. As a result, the variation of the diagonal resistance may be caused by the quasi-particle tunneling.

### Two-terminal conductance

**Fig. 4:**
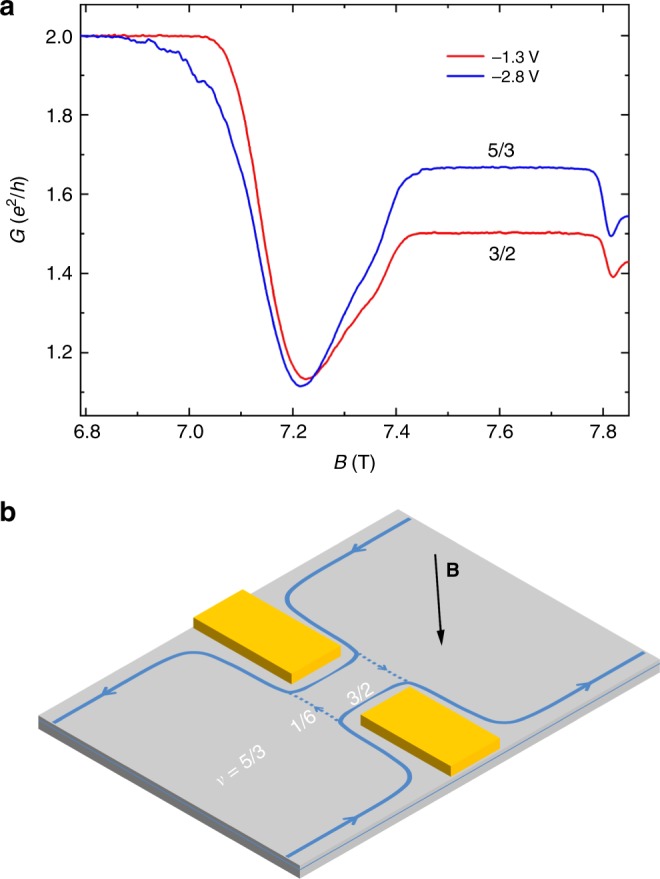
Gate voltage dependence of the two-terminal conductance and the sketch of the propagating edge channels. **a** Two-terminal conductance traces at 18 mK with *V*_gate_ = −1.3 V and *V*_gate_ = −2.8 V respectively. The measurement was carried out by applying a voltage excitation to the contact *S* and measuring the current from contact *D* (contacts *S* and *D* are the same configurations as that shown in Fig. 1a). **b** The sketch of the propagating edge channels for the formation of the 3/2 FQH plateau. In the bulk, the filling factor is 5/3. In the confined region, the straight lines represent the transmitting conductance quantized at $\frac{3}{2}\frac{{e^2}}{h}$, and the dashed lines represent the tunneling conductance quantized at $\frac{1}{6}\frac{{e^2}}{h}$. Source data are provided as a Source Data file

From the Landauer-Büttiker theory^26^, when the diagonal resistance develops the 3/2 FQH plateau, the conductance $\frac{3}{2}\frac{{e^2}}{h}$ will transmit through the confined region, which has been verified in our measurements. Fig. 4a shows the two-terminal conductance traces at 18 mK. At filling factor 5/3, the conductance is quantized at $\frac{5}{3}\frac{{e^2}}{h}$ with *V*_gate_ = −2.8 V and at $\frac{3}{2}\frac{{e^2}}{h}$ with *V*_gate_ = −1.3 V. The conductance in the bulk of the Hall bar should be $\frac{5}{3}\frac{{e^2}}{h}$ at filling factor 5/3. When the conductance transmitting the confined region is $\frac{3}{2}\frac{{e^2}}{h}$, the tunneling conductance will be $\frac{1}{6}\frac{{e^2}}{h}$. The corresponding propagating edge channels are sketched in Fig. 4b, where the dashed lines represent the tunneling conductance quantized at $\frac{1}{6}\frac{{e^2}}{h}$. This edge channel picture has also been verified by four-terminal resistance and two-terminal conductance measurements with the reversed magnetic field (Supplementary Figs. 4 and 5).

## Discussion

In general, the density of the gate-defined confinement region is smaller than that of the bulk, and affected by the gate voltage. However, the gate annealing procedure can keep uniform density for the whole sample in our measurements. This can be verified by the magnetic field dependence of the IQH and FQH plateaus in Hall traces in Fig. 1c and Supplementary Fig. 6. By comparing the diagonal resistance and the Hall resistance as a function of magnetic field for the gating condition of developing the 3/2 FQH plateau (Fig. 3a), the 3/2 plateau in this measurement appears at the filling factor of 5/3. What’s more, if the density of the confinement is reduced to filling factor 3/2 when the 3/2 plateau shows up at −1.8 V (Fig. 3a), then further negatively charging the top gate will only maintain or keep reducing the density in the confinement. As a result, the 5/3 plateau could not show up anymore at the filling factor 3/2 or at a smaller filling factor, which is contradicted to the experimental observation (−2.8 V at Fig. 3a). This argument serves as an additional clue that the 3/2 FQH plateau appears at the filling factor of 5/3. The 3/2 FQH plateau has also been observed in Charles M. Marcus group, shown in Yiming Zhang’s thesis^27^. In their measurements, the 3/2 FQH plateaus appeared in samples with both uniform density and non-uniform density. From the corresponding *R*-*B* features between *R*_D_ and *R*_XY_ in Fig. 1c and Supplementary Fig. 6, the 3/2 FQH plateau we observed is unlikely induced only by the density variation in the confined region.

Disorder can drive the collapse of energy gap and then induce phase transition from the FQH state to another state^28,29^. At *ν* = 5/3 FQH state, the edge state could be complicated due to potential edge reconstruction and neutral mode^20,23,30^. The formation of the 3/2 FQH plateau could be caused by a more complicated edge reconstruction with lateral confinement at *ν* = 5/3. In our system, the disorders induced by the residual potential in the confined region could also help with the collapse of the 5/3 FQH state and the formation of intriguing edge structure and excitation with fractional charge. The 3/2 FQH plateau has been found in the single-layer two-dimensional system of ZnO where Landau level crossing induced by tilted magnetic field is needed^10^, while there is no in-plane magnetic field in our measurements. The exact origin of the 3/2 FQH plateau remains an open theoretical question to be investigated.

## Methods

### Sample fabrication

The samples were made from a wafer of GaAs/AlGaAs heterostructures. The center of the 30 nm quantum well is 210 nm below the surface, with modulated Si doping at ~100 nm below and above the quantum well center. The mobility is higher than 1.0 × 10^7^ cm^2^ V^−1^ s^−1^ at 20 mK. A 150 μm wide Hall bar was shaped by wet etching. Ohmic contacts were made of Pt/Au/Ge alloy deposited by e-beam evaporation and annealed at 550 °C for 100 s. Two separate Cr/Au gates were deposited on the surface of the sample to form the confined region. In this experiment, samples with different confined region sizes were fabricated and measured. The dimensions of these geometries are 1 × 2 μm^2^, 0.3 × 2 μm^2^ and 1.5 × 3 μm^2^, respectively. The confined region on the samples used in the main text is the size of 1 × 2 μm^2^. The data from other samples are shown in the Supplementary Figs. 1–3.

### Measurement techniques

The measurements were carried out in a dilution refrigerator with a base refrigerator temperature below 6 mK. The temperatures given in this work are electron temperatures. In the four-terminal resistance measurements, the Hall resistance *R*_XY_ and the diagonal resistance *R*_D_ were measured simultaneously using lock-in techniques at 6.47 Hz with 1 nA. The two-terminal conductance measurements were also carried out using lock-in techniques at 6.47 Hz with voltage excitations smaller than 35 μV.

## Supplementary information

Supplementary Information

Source Data

## Data Availability

The authors declare that the main data supporting the findings of this study are available within the article and its Supplementary Materials. Extra data are available from the corresponding author upon request. The source data underlying Figs. 1b, c, 2, 3, 4a and Supplementary Figs. 1–6 are provided as a Source Data file.
