# Supplementary material for: 3/2 fractional quantum Hall plateau in confined two-dimensional electron gas
Source: Nat Commun. 2019 Sep 25;10:4351. doi: 10.1038/s41467-019-12245-y (PMC6761136; doi:10.1038/s41467-019-12245-y)
Supplement: Supplementary file 1 — Supplementary Information [file 41467_2019_12245_MOESM1_ESM.pdf]

Supplementary Information

“3/2 Fractional Quantum Hall Plateau in Confined Two-Dimensional Electron Gas”

Fu et al.

Supplementary Note 1: Experimental results

This section shows more experimental results measured in different samples. Data in Supplementary Figure 1, Figure 2 and Figure 3 come from three more samples, and the appearance of the 3/2 FQH plateau is consistent with the results in the main text. Influence of the annealing procedure on the 3/2 FQH plateau is shown Supplementary Figure 2.

Supplementary Figure 4 and Figure 5 show the results measured in the opposite magnetic field directions. The diagonal resistance R_{D} in our experimental setup is determined by the voltage difference between contacts 3 and 4, explicitly as $R_{\mathrm{D}} = \frac{V_3 - V_4}{I_{\mathrm{AC}}}$ (Fig. 1 in the main text). Therefore, $R_{\mathrm{D}} = \frac{V_3 - V_6}{I_{\mathrm{AC}}} + \frac{V_6 - V_4}{I_{\mathrm{AC}}} = R_{\mathrm{L}} + R_{\mathrm{XY}}$; the observation that the diagonal resistance R_{D} is larger than the Hall resistance R_{XY} indicates that part of the edge channel does not pass through the confined region (Fig. 4b in the main text). If we keep the same measurement setup and reverse the directions of the edge channel by switching the magnetic field, we should measure R_{L}-$|R_{\mathrm{XY}}|$. Now the sign of R_{XY} will be changed by the magnetic field while the sign of R_{L} will remain the same because the source and drain remain the same positions. Because V_3 is smaller than V_4 with a reversed magnetic field, the measured diagonal resistance R_{D} should be also negative. Experimentally, we did observe a new plateau quantized at negative $\left(\frac{h}{e^2}\right)/\left(\frac{15}{8}\right)$ with the same experimental setup and opposite magnetic field, as shown in Supplementary Figure 4. The average of resistance $\left(\frac{h}{e^2}\right)/\left(\frac{3}{2}\right)$ and resistance $\left(\frac{h}{e^2}\right)/\left(\frac{15}{8}\right)$ is $\left(\frac{h}{e^2}\right)/\left(\frac{5}{3}\right)$, which is identical to R_{XY}, as expected in the picture of edge channel. The observation of the -15/8 FQH plateau verifies the edge channel reflection in the confined region.

Supplementary Figure 6 shows the Hall resistance and the diagonal resistance traces with different gate voltages to demonstrate the nearly uniform density in the whole sample. In general, more negative gate voltage will cause lower density in the confined region. As shown in Supplementary Figure 6a, if the gates have never been annealed, more negative gate voltages result in larger slopes of the diagonal resistance traces, corresponding to lower densities in the confined region. And the diagonal resistance traces with different densities develop with the same IQH plateaus at different magnetic fields. As shown in Supplementary Figure 6b, when the gates have been annealed with -4.5 V, the slopes of the diagonal resistance traces with less negative gate voltages are larger than that with -4.5 V, and no well-defined IQH plateaus appear, which may be caused by edge current backscattering rather than the density difference. If we extract the density from the slopes at -1.3 V from Supplementary Figure 6b between 0.15 T and 0.35 T, and calculate the filling factors at high magnetic field, then the $\nu = 2$ state unreasonably lies between filling factor 1.9 and 1.55, as shown in Supplementary Figure 6c. In addition, the 3/2 plateau doesn't match the filling factor 3/2 even from the density calculated from the low field data (red top x-axis). We believe the density in the whole sample is nearly uniform.

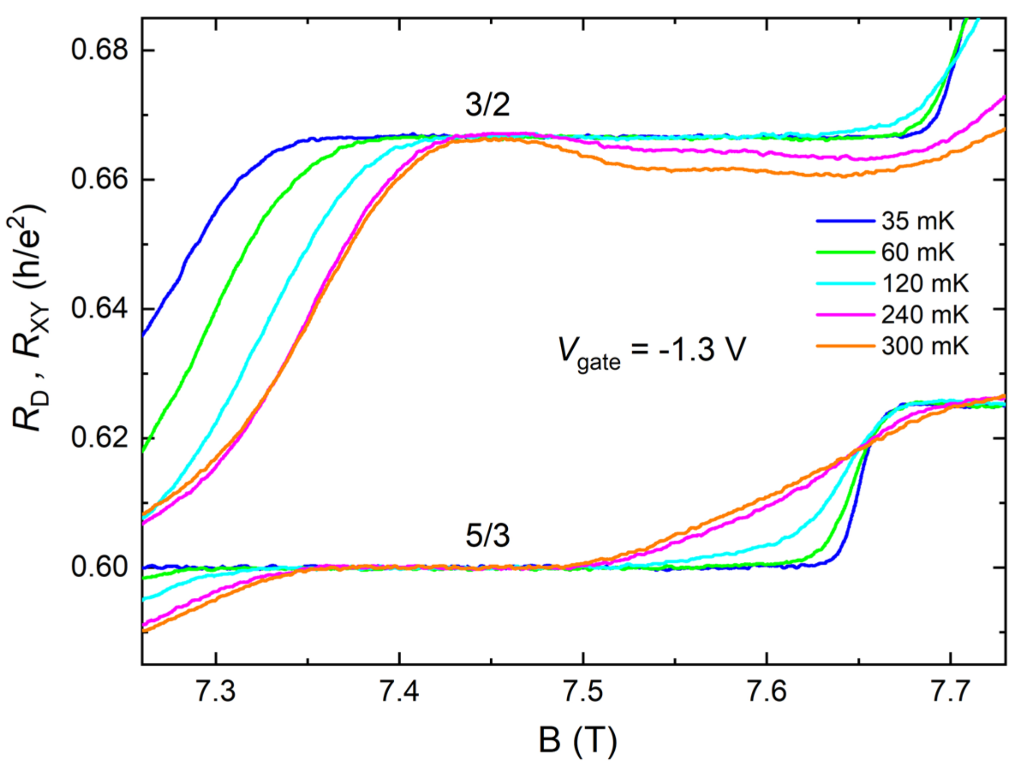

Supplementary Figure 1. The diagonal resistance and the Hall resistance traces around the 5/3 FQH state with V_{gate} = -1.3 V at different temperatures. The widths of the 3/2 and 5/3 FQH plateaus decrease with increasing temperature, and the 3/2 FQH plateau can even survive up to 300 mK. Data are from another sample with the same confined region as that used in the main text. Source data are provided as a Source Data file.

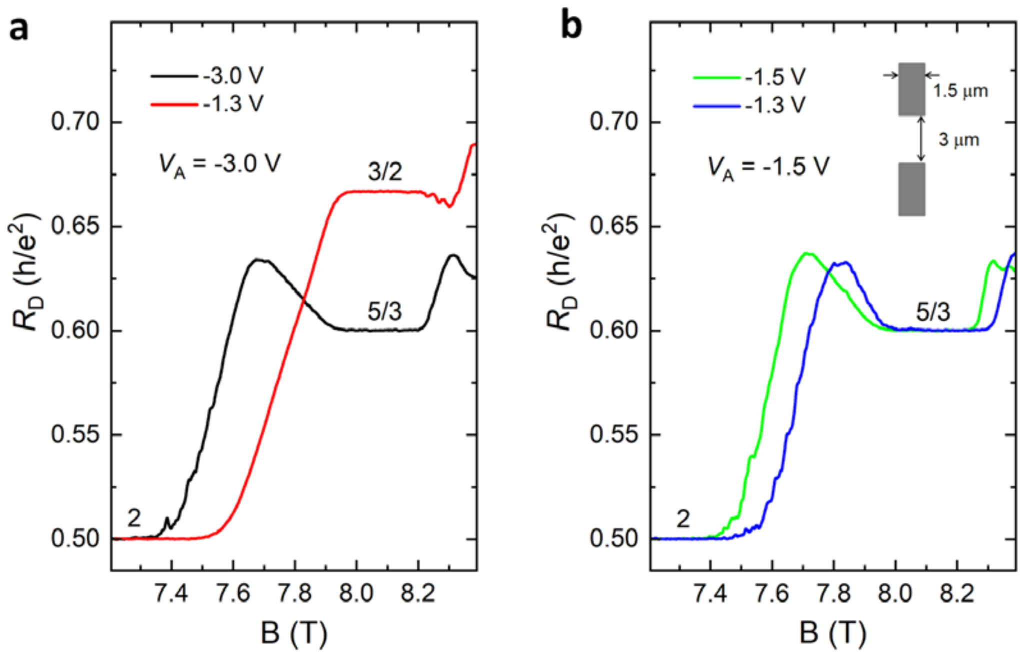

Supplementary Figure 2. Influence of the annealing procedure on the 3/2 FQH plateau. a, The gates were annealed at -3.0 V. The diagonal resistance develops the 5/3 FQH plateau with V_{gate} = -3.0 V and the 3/2 FQH plateau with V_{gate} = -1.3 V. **b,** The gates were annealed at -1.5 V. The diagonal resistance only develops the 5/3 FQH plateau with both V_{gate} = -1.5 V and V_{gate} = -1.3 V. The confined region of this device is shown in the inset of **b** as a sketch. Source data are provided as a Source Data file.

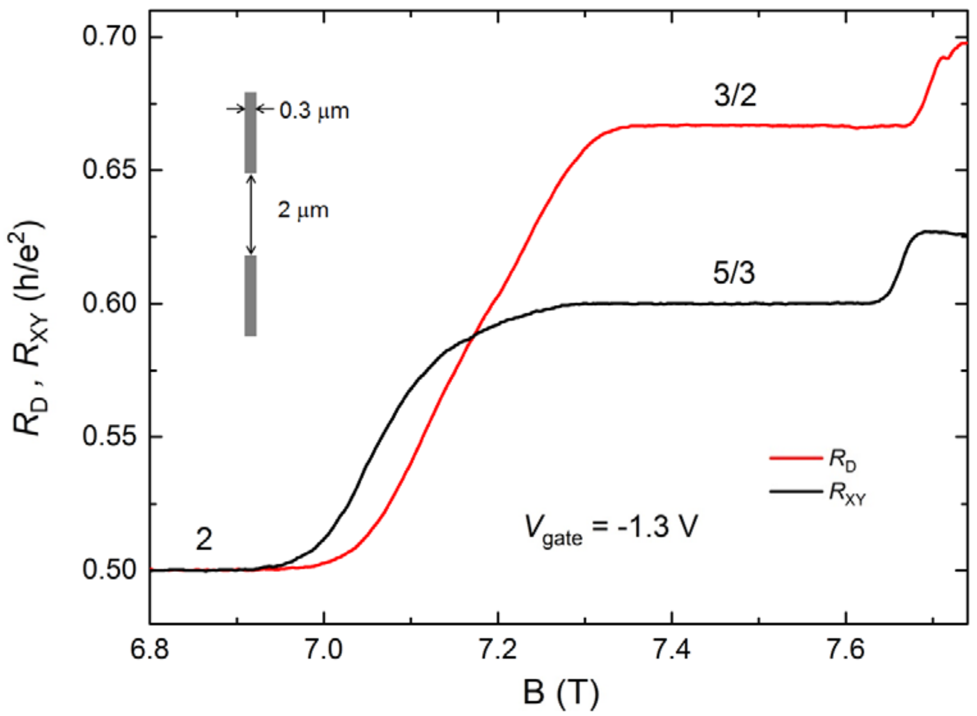

Supplementary Figure 3. The diagonal resistance and the Hall resistance traces with V_{gate} = -1.3 V at 35 mK from another device. The gates were annealed at -4.5 V. The diagonal resistance develops the 3/2 FQH plateau and the Hall resistance develops the 5/3 FQH plateau. The inset is a sketch of the confined region. Source data are provided as a Source Data file.

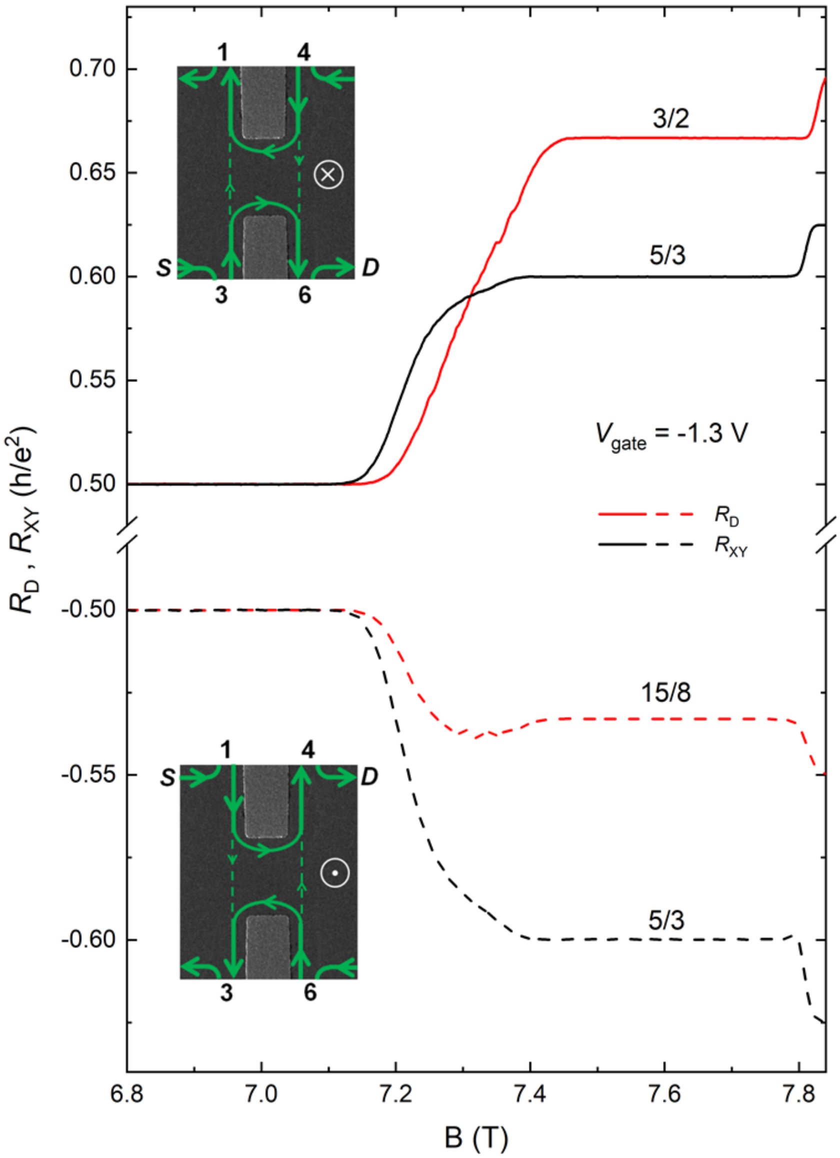

Supplementary Figure 4. The diagonal resistance and the Hall resistance traces in opposite magnetic field directions with V_{gate} = -1.3 V at 18 mK. R_D was measured from contact 3 to contact 4 and R_{XY} was measured effectively as from contact 6 to contact 4. When the magnetic field direction is into the page (the upper inset), R_{XY} is quantized at $\left(\frac{h}{e^2}\right)/\left(\frac{5}{3}\right)$ (solid black line) and R_D is quantized at $\left(\frac{h}{e^2}\right)/\left(\frac{3}{2}\right)$ (solid red line). When the magnetic field direction is out of the page, only the propagating directions of the edge channels are reversed (the lower inset). As a result, R_{XY} is quantized at $-\left(\frac{h}{e^2}\right)/\left(\frac{5}{3}\right)$ (dashed black line) and R_D is quantized at $-\left(\frac{h}{e^2}\right)/\left(\frac{15}{8}\right)$ (dashed red line). Data are from the same sample used in the main text. Source data are provided as a Source Data file.

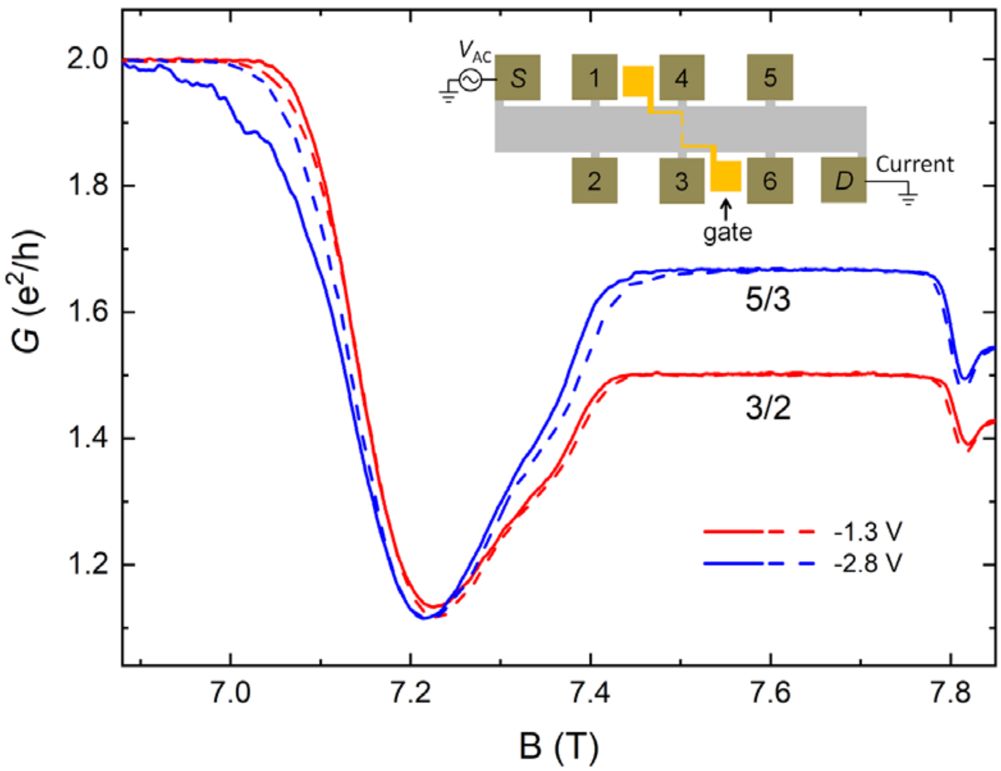

Supplementary Figure 5. Two-terminal conductance traces in opposite magnetic field directions at 18 mK. The conductance was obtained by applying a voltage excitation to the contact *S* and measuring the current from contact *D*. Solid lines and dashed lines represent data measured in the opposite magnetic field directions respectively. The inset is the sketch of the Hall bar and the measurement setup. The observation of the 3/2 FQH conductance plateau in both field directions verifies the edge channel reflection picture in the confined region. Data are from the same sample used in the main text. Source data are provided as a Source Data file.

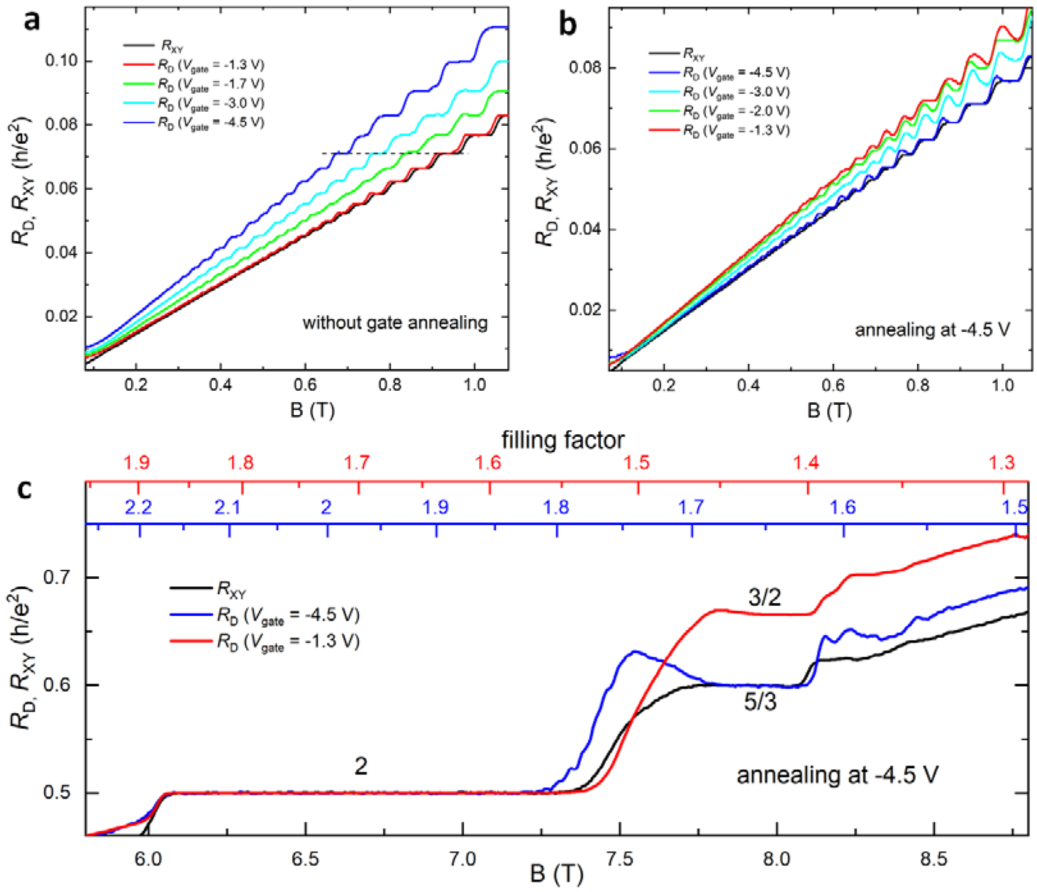

Supplementary Figure 6. The diagonal resistance and the Hall resistance traces with different gate voltages. a, The diagonal resistances and the Hall resistance traces at low magnetic field without gate annealing. The horizontal dashed line is an eye-guide to show that the same IQH plateau appears with different electron densities. **b,** The diagonal resistances and the Hall resistance traces at low magnetic field with gate annealing at -4.5 V. **c,** The diagonal resistances and the Hall resistance traces at high magnetic field with gate annealing at -4.5 V. Top red (blue) axis shows the filling factors calculated from the diagonal resistance traces with -1.3 V (-4.5 V) at low magnetic field from **b**. The difference in *R*-B traces at low field should result from confinement effect but not the density difference, because the $\nu = 2$ state at V_{gate} = -1.3 V should not lie between filling factors 1.9 and 1.55. Source data are provided as a Source Data file.

Supplementary Note 2: Preliminary theoretical attempt

A tentative explanation to the origin of the 3/2 FQH plateau could be a further 1/2 quantization of the quasi-particles with e/3 fractional charge in the 5/3 FQH state, which causes a $\frac{1}{6}\frac{e^2}{h}$ backscattering and therefore the 3/2 quantum Hall plateau. Such 1/2 quantization is proposed to be related to the half-charged Jackiw-Rebbi zero-mode [1] induced by a topological phase transition near the confined geometry.

A heuristic description could be that, in the perspective of composite fermion, as an illustration, the edge channels of the ν = 5/3 FQH state can be treated as containing two downstream e^* = e/3 charged edge channels with different chemical potentials, moving in the presence of the residual magnetic field. A one-dimensional four-band Hamiltonian [2] can be constructed in the basis of these e^* = e/3 edge channels as (R_+, L_-, R_-, L_+) (where R/L for right-/left-moving channel in the opposite edges, and $\pm$ for edge channels with higher/lower chemical potential, respectively) as $H = H_0 + \Delta H$. Here $H_0 = \hat{p}_x\tau_0\rho_3 + t\tau_1\rho_1 + \mu\tau_3\rho_3$ includes the linearized kinetic energy term $\hat{p}_x$, inter-edge tunneling term t, and chemical potential μ [τ_0 is identity matrix and $\tau_{i=1,2,3}$ are Pauli matrices acting on the $(R_+/L_-, R_-/L_+)^T$ spinor, and ρ-matrices are Pauli matrices working in the right-/left-moving space], while $\Delta H = \Delta \cdot \tau_0\rho_1$ is a perturbative (Δ is small but non-zero) coupling term between channels with different chemical potentials.

The dispersions for the unperturbed four-band Hamiltonian H_0 are: $\pm\mu \pm \sqrt{k_x^2 + t^2}$. For non-zero t, the uppermost band $|\Psi_u\rangle = C\left(1,0,0, -k_x/t + \sqrt{k_x^2+t^2}/t\right)^T$ and the lowermost band $|\Psi_l\rangle = C\left(0,1, k_x/t - \sqrt{k_x^2+t^2}/t, 0\right)^T$ (C is the normalization constant) are always gapped (see Supplementary Figure 7), therefore they are not involved in the topological phase transition and contribute $\frac{1}{3}\frac{e^2}{h}$ Hall conductance as ever. However, for the intermediate two bands

$E_\pm = \mp\mu \pm \sqrt{k_x^2 + t^2}$ with $|\Psi_-\rangle = C\left(k_x/t - \sqrt{k_x^2+t^2}/t, 0,0,1\right)^T$ and $|\Psi_+\rangle = C\left(0, -k_x/t + \sqrt{k_x^2+t^2}/t, 1,0\right)^T$, projecting ΔH onto $(|\Psi_-\rangle, |\Psi_+\rangle)^T$ leads to an effective Hamiltonian equivalent to one-dimensional topological insulator:

$$H_{\text{eff}} = \Delta \frac{k_x}{\sqrt{k_x^2 + t^2}}\sigma_x + \left(\mu - \sqrt{k_x^2 + t^2}\right)\sigma_z \tag{1}$$

where the σ-matrices are Pauli matrices acting on the higher-/lower-chemical potential spinor. The competition between the inter-edge tunneling strength t and the chemical potential μ leads to a topological phase transition (see Supplementary Figure 7). At the domain wall separating the tunneling-dominated region ($t > \mu$; near the confined geometry) and the magnetic-field-dominated region ($t < \mu$; far away from the confined geometry), there are topologically protected Jackiw-Rebbi type bound states possessing $e^*/2$ = e/6 charge [1, 2].

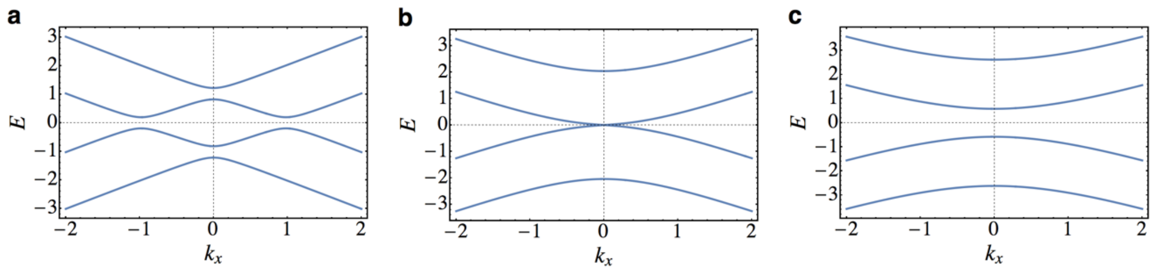

Supplementary Figure 7. Dispersions and topological phase transition of the one-dimensional four-band Hamiltonian $H = H_0 + \Delta H$. a, Magnetic-field-dominated region ($t < \mu$). $\mu = 1, \Delta = 0.2$, and $t = 0.2$. **b,** Phase transition point ($t \approx \mu$). $\mu = 1, \Delta = 0.2$, and $t = 1.02$. **c,** Tunneling-dominated region ($t > \mu$). $\mu = 1, \Delta = 0.2$, and $t = 1.6$. The uppermost and the lowermost bands are always gapped, while the intermediate two bands can be approximately treated as a one-dimensional topological insulator. The Jackiw-Rebbi type zero-modes are bounded at the domain wall separating these two topologically distinct phases (**a** and **c**).

However, compared with the two-dimensional geometry of the experimental setup, the model described above is actually a quasi-one-dimensional one. There could be several ways to generalize the Jackiw-Rebbi zero-mode possessing half charge $e^*/2$ to the two-dimensional case. Two-dimensional $e^*/2$ zero-mode could be realized by introducing a vortex [3, 4]. However, such an $e^*/2$ zero-mode is bound state uninvolved in the electrical transport except a charge pump is provided [5]. Another proposed generalization could be the Moore-Read state [6, 7]. Here, in this two-dimensional electron system, we suppose that a promising two-dimensional generalization of the Jackiw-Rebbi type zero-mode could be the massless Dirac fermion, which is analogous to the generalization from the localized end-estate (in one-dimensional topological insulator) to the edge states with linear dispersion (in two-dimensional topological insulator). Such generalization is also consistent with Son's theory [8] that the composite fermions in the half-filled Landau level with particle-hole symmetry are massless Dirac fermions. In the presence of the residual magnetic field, the massless Dirac fermions' zeroth Landau level forms edge channel carrying half quantized Hall conductance $\frac{1}{2} \cdot \frac{1}{3}\frac{e^2}{h}$ [9], which is analogous to the half-charged bound state in the one-dimensional case. Consequently, such an edge channel with $\frac{1}{6}\frac{e^2}{h}$ conductance in the tunneling-dominated region, may accounts for the backscattering conductance and therefore explains the emergence of the $\frac{3}{2}\frac{e^2}{h}$ Hall plateau.

Such a heuristic model is stated here as an illustration of the proposed theoretical scheme. The actual edge states of the 5/3 FQH state could be much more complicated due to edge reconstruction, such as containing two downstream $e^* = e/3$ charged edge channels as well as two upstream neutral charge modes [10, 11]. Generally, the inter-edge tunneling strengths are different for different edge channels, which are relevant to the spatial distribution of the edge

channels' wave-functions. Besides, in realistic experimental device, the inter-edge tunneling strength could be spatially inhomogeneous, so that the tunneling may also transfer quasiparticles between opposite edges and therefore induce a position dependence of the chemical potential. Moreover, the free fermion description of the fractionally-charged edge channels is also inaccurate. For these reasons and the different possibilities of the generalization of Jackiw-Rebbi zero-mode mentioned above, the theoretical descriptions here are provided as a tentative hypothesis. A solid proof of the origin of such a novel 3/2 FQH state still remains as an open question to be further investigated.

Supplementary references

1. Jackiw R, Rebbi C. Solitons with fermion number 1/2*. *Phys. Rev. D* **13**, 3398-3409 (1976).
2. Klinovaja J, Loss D. Fractional charge and spin states in topological insulator constrictions. *Phys. Rev. B* **92**, 121410 (2015).
3. Hou C-Y, Chamon C, Mudry C. Electron Fractionalization in Two-Dimensional Graphenelike Structures. *Phys. Rev. Lett.* **98**, 186809 (2007).
4. Seradjeh B, Weeks C, Franz M. Fractionalization in a square-lattice model with time-reversal symmetry. *Phys. Rev. B* **77**, 033104 (2008).
5. Qi X-L, Hughes TL, Zhang S-C. Fractional charge and quantized current in the quantum spin Hall state. *Nat. Phys.* **4**, 273-276 (2008).
6. Moore G, Read N. Nonabelions in the fractional quantum hall effect. *Nucl. Phys. B* **360**, 362-396 (1991).
7. Lee D-H, Zhang G-M, Xiang T. Edge Solitons of Topological Insulators and Fractionalized Quasiparticles in Two Dimensions. *Phys. Rev. Lett.* **99**, 196805 (2007).
8. Son DT. Is the Composite Fermion a Dirac Particle? *Phys. Rev. X* **5**, 031027 (2015).
9. Xu Y, *et al.* Observation of topological surface state quantum Hall effect in an intrinsic three-dimensional topological insulator. *Nat. Phys.* **10**, 956-963 (2014).
10. Bid A, *et al.* Observation of neutral modes in the fractional quantum Hall regime. *Nature* **466**, 585-590 (2010).
11. Sabo R, *et al.* Edge reconstruction in fractional quantum Hall states. *Nat. Phys.* **13**, 491-496 (2017).
